# Unlocking Performance: Pharmacogenomic Insights for Athletes

**DOI:** 10.1055/a-2957-1103

**Published:** 2026-09-28

**Authors:** Kristal S. Potter, Jessica Beal-Stahl, Megan Luaces, Kevin Chin, Anam F. Ismail, Courtney Guerra

**Affiliations:** 1 Department of Clinical and Administrative Sciences 535884 Larkin University College of PharmacyMiamiFloridaUnited States; 2 Clinical ResearchSports Pharmacy NetworkParklandFloridaUnited States

**Keywords:** pharmacogenomics, pharmacogenetics, sports pharmacy, genetics

## Abstract

Pharmacogenomics is the study of how genetic differences influence drug responses, offering a safer and more effective prescribing model. While pharmacogenomics is well-established in specialties such as oncology and psychiatry, its application in sports medicine remains limited. Athletes frequently use medications such as nonsteroidal anti-inflammatory drugs, opioids, and selective serotonin reuptake inhibitors, and these medications have well-documented gene–drug interactions. This review explores pharmacogenomics considerations for these commonly used substances in athletic populations, emphasizing how genetic variability in drug-metabolizing enzymes, including CYP2C9, CYP2D6, and CYP2C19, can impact medication efficacy and side effect risks. We summarize current evidence, highlight clinical guidelines, and outline how pharmacogenomics-guided prescribing could improve athlete care. By integrating pharmacogenomics into sports medicine, clinicians can tailor medication strategies to each athlete’s genetic profile, supporting both health and performance. Broader adoption will require increased clinician education, interdisciplinary collaboration, and research specific to athletic populations, but pharmacogenomics offers a promising tool for advancing individualized, precision-based athlete healthcare.

## Introduction

Pharmacogenomics (PGx) is the study of how genetics can affect individual responses to medications. 1​ Genetic variations can significantly influence drug responses, from life-threatening adverse reactions to no therapeutic effects. PGx testing is emerging as a crucial element of patient-centered care and individualized medicine. By identifying actionable variants in the genes encoding drug-metabolizing enzymes, drug transporters, and drug targets, healthcare providers can optimize drug selection and dosing.

### Genetic polymorphisms in metabolizing enzymes

Variations in cytochrome P450 (CYP450) enzymes, caused by genetic polymorphisms, can significantly alter drug metabolism. Metabolism genes can be classified into different phenotypes: poor metabolizer (PM), intermediate metabolizer (IM), normal metabolizer (NM), rapid metabolizer (RM), or ultrarapid metabolizer (UM). 2​ These classifications correspond to varying levels of enzyme activity, from reduction or absent activity in PMs to significantly increased activity in UMs ( Fig. 1 ). These enzymatic differences can directly impact the rate and extent of drug metabolism. In clinical practice, this variability may result in subtherapeutic drug concentrations, increased risk of adverse events, or treatment failure, underscoring the clinical utility of PGx testing in individualized medicine. Genetic variations can also extend to drug transporters and drug targets, which can significantly influence the pharmacokinetics (PK) and pharmacodynamics (PD) of medications. 3​ PGx testing enables clinicians to tailor medication selection and dosages based on a patient’s genetic profile, optimizing therapeutic efficacy while minimizing adverse drug reactions (ADRs) and side effects.

**Fig. 1:**
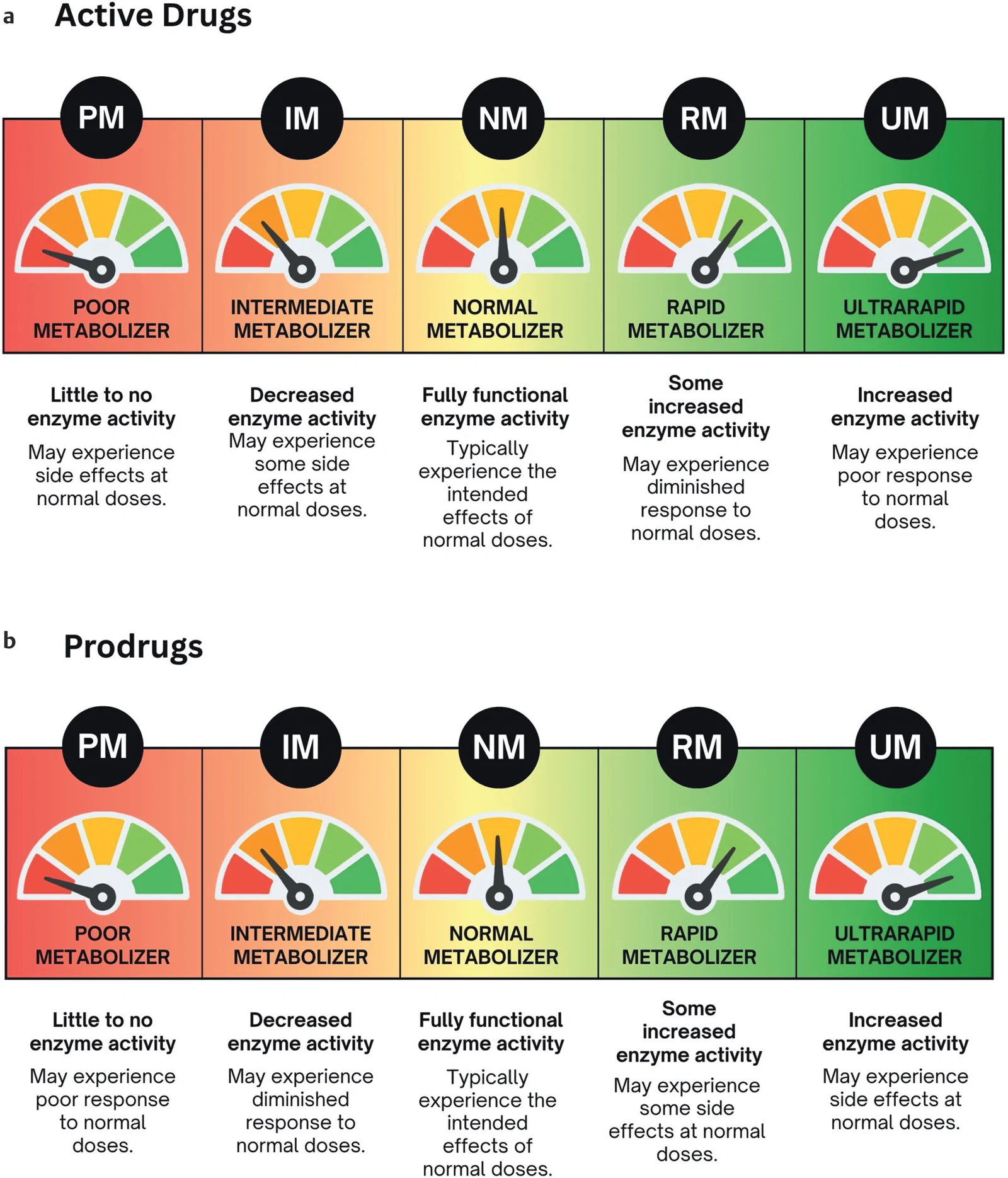
Metabolizer types: Visual representation of the five major CYP450 metabolizer phenotypes: PM, IM, NM, RM, and UM. These phenotypes influence the rate at which individuals metabolize medications, with implications for drug efficacy, safety, and dosing. ( **a** ) For active drugs, decreased enzyme activity may increase drug exposure and adverse-effect risk, whereas increased enzyme activity may reduce drug exposure and therapeutic response. ( **b** ) For prodrugs requiring metabolic activation, decreased enzyme activity may reduce active metabolite formation and therapeutic response, whereas increased enzyme activity may increase active metabolite formation and toxicity risk. IM, intermediate metabolizer; NM, normal metabolizer; PM, poor metabolizer; RM, rapid metabolizer; UM, ultrarapid metabolizer.

### Pharmacogenomics in clinical practice

PGx is increasingly being integrated into clinical practice, particularly in oncology, psychiatry, and cardiology practices. An analysis of 487,409 participants in the United Kingdom revealed that, on average, individuals may have an atypical response to 10.3 medications, and nearly 24% had been prescribed at least one drug to which they were predicted to have an atypical response. 4​ Similarly, a study of 43,359 patients found that 98.9% carried one or more PGx actionable alleles, suggesting that treatment modification would be recommended. 5​ Of these patients, 14.2% ( *n* =6,157) had been prescribed medications for which their genetic profile could impact drug responses. To support clinical application, organizations such as the Clinical Pharmacogenetics Implementation Consortium (CPIC) and the Dutch Pharmacogenetics Working Group (DPWG) have developed clinical guidelines and evidence-based tools to help healthcare providers interpret the genetic test results and make clinical decisions based on the results. 6​

### Pharmacogenomics in sports medicine

Despite significant advances in the accessibility and clinical integration of PGx, the implementation of PGx testing remains limited in certain fields, most notably sports medicine. A comprehensive literature review found few published studies and minimal evidence supporting the use of PGx-guided prescribing in athletic populations. This gap in research is concerning, given the unique physiological demands, altered PK, and frequent medication use among athletic populations. 7​ ​8​

PGx provides an important framework for predicting variability in drug response; however, in sports medicine, genotype alone is insufficient to characterize PK and PD in athletes. A medication’s therapeutic effects occur within a highly dynamic physiological environment. Training status, hydration, exercise-induced changes in organ perfusion, body composition, inflammatory signaling, nutritional status, and even supplement use can influence drug response. 9​ 10​ ​11​ A 2024 perspective on PGx suggests that integrating PGx with other “omics” may better capture changes in drug response associated with environmental exposures and physiological stress. 12​

Athletes may also experience periods of heightened systemic and local inflammation and physiological stress related to high training loads, competition, travel, injury, and illness. Recent work on “phenoconversion” further demonstrates that non-genetic influences, including inflammation and physiological stress, may shift the phenotype away from what PGx tests predict. 13​

Nutritional challenges common in sports, including low energy availability, micronutrient depletion, restrictive dietary patterns, and use of supplements or ergogenic aids, add complexity through drug–food–gene and drug–supplement–gene interactions. Supplements that induce or inhibit CYP enzymes or drug transporters may alter drug exposure even when﻿ prescribing follows PGx guidelines. 14​ Therefore, PGx testing in athletes should be interpreted within the broader context of physiology, training load, inflammatory state, nutrition, recovery status, and environmental exposures.

The potential role of genetic variability in shaping how athletes respond to common medications has largely been overlooked. Athletes are routinely prescribed or self-administer medications to address a range of conditions, including pain, inflammation, mental health disorders, fatigue, and other health conditions. As precision medicine continues to evolve, the inclusion of PGx in sports medicine holds potential as a valuable tool to individualize medication strategies, enhancing efficacy, minimizing ADRs, and supporting performance optimization and athlete safety. 8​

PGx may also affect anti-doping testing and therapeutic use exemption considerations. For example, CYP2D6 polymorphisms can alter codeine metabolism and influence morphine-to-codeine metabolite ratios. 15​ This may affect the interpretation of opioid-related doping analyses and complicate the differentiation between the therapeutic use and the prohibited substance use in athletes. 16​ UMs of codeine may have increased morphine formation despite the appropriate therapeutic codeine use. Another example is caffeine metabolism, where genetic differences in CYP1A2 activity can significantly influence the caffeine metabolism and clearance. 17​ In athletic organizations that monitor urinary caffeine concentrations, such as the NCAA, genetic variability could theoretically contribute to differences in caffeine levels among athletes consuming comparable amounts.

This review aims to bridge the gap between PGx and sports medicine by examining gene–drug interactions relevant to medications commonly used by athletes. We focused on drug classes commonly used in athletic populations, including selective serotonin reuptake inhibitors (SSRIs), serotonin–norepinephrine reuptake inhibitors (SNRIs), opioids, nonsteroidal anti-inflammatory drugs (NSAIDs), and caffeine. We aimed to summarize the current understanding of gene–drug interactions and highlight the potential role of PGx in supporting individualized medication management for athletes.

## Methods

Our objective was to synthesize current evidence on gene–drug interactions relevant to sports medicine and performance-related outcomes. A narrative literature review was conducted between January and June 2025 using the peer-reviewed literature, PGx databases, and clinical guideline resources. Medication classes were selected through a consensus-driven process among the authors based on clinical experience, published literature, and commonly prescribed or utilized medications in athletic populations. 7​ 18​​ 19​ ​20​

An initial list of medications frequently encountered in sports medicine and athlete care was generated, including agents used for pain management, mental health conditions, and performance-related purposes. Medications were subsequently screened for the availability of clinically relevant PGx evidence. The final selection focused on three major therapeutic categories: analgesics (NSAIDs and opioids), psychiatric medications (SSRIs and SNRIs), and caffeine.

For each medication, relevant PGx data were identified through the review of the ClinPGx database (formerly PharmGKB), CPIC guidelines, and supporting peer-reviewed literature. Data extraction focused on clinically actionable gene–drug interactions involving major CYP450 enzymes, including CYP2C9, CYP2C19, and CYP2D6 ( Fig. 2 ). We reviewed gene variants, metabolizer phenotypes, clinical annotations, and therapeutic recommendations where available.

**Fig. 2:**
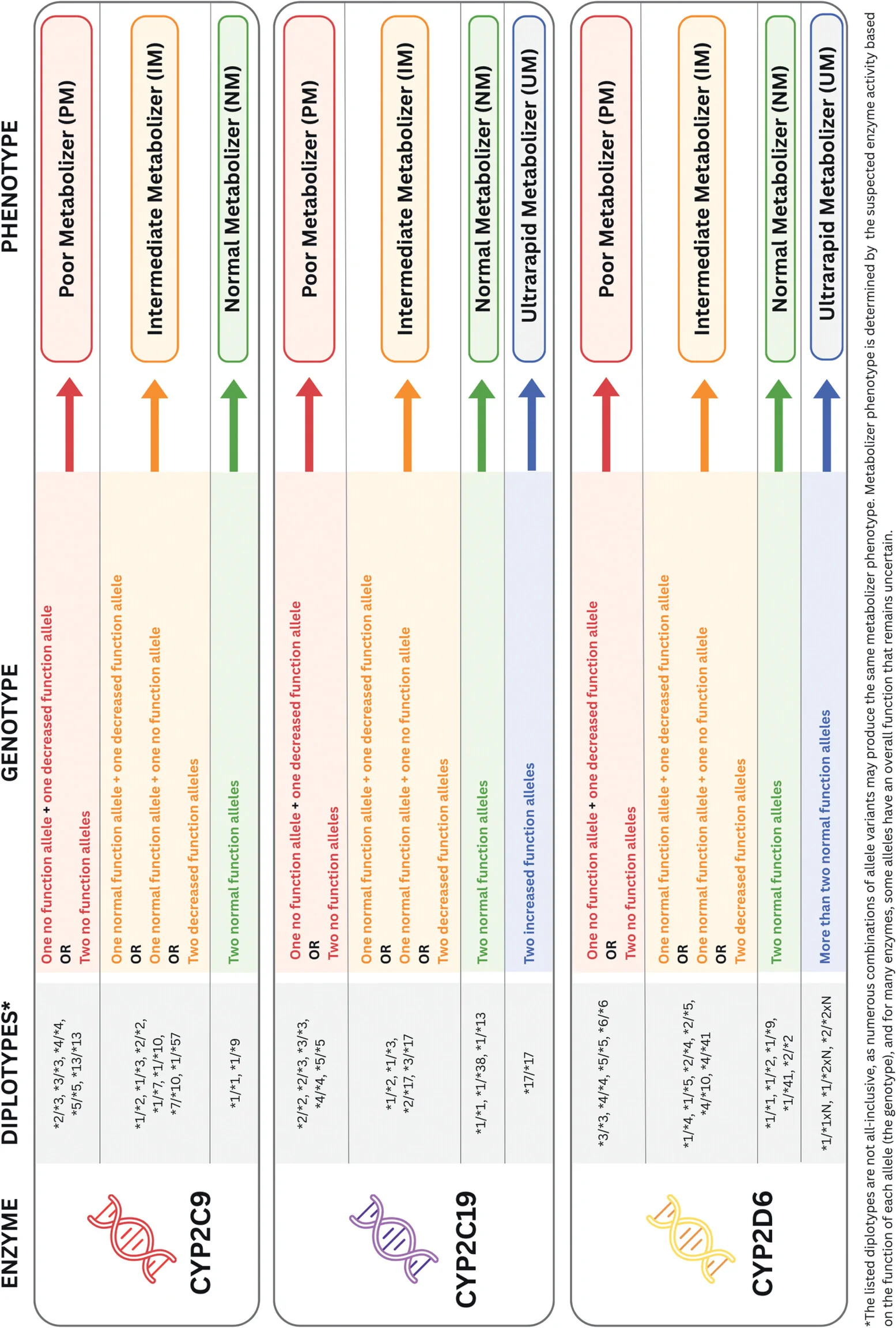
Genotype-based metabolizer phenotype classification for selected enzymes: Representative genotype patterns are grouped according to predicted enzyme activity and corresponding metabolizer status, including PM, IM, NM, and UM phenotypes. Phenotype assignment is based on the combined functional activity of inherited alleles. IM, intermediate metabolizer; NM, normal metabolizer; PM, poor metabolizer; UM, ultrarapid metabolizer.

Additional literature was identified through the manual review of reference lists and sports medicine consensus statements to provide the clinical context regarding medication utilization in athletic populations. Emphasis was placed on studies and reviews involving athlete populations, sports medicine practice, medication safety, and performance-related considerations. The resulting evidence was synthesized narratively to summarize clinically relevant PGx implications for medications commonly used in athletes ( Table 1 ). PGx evidence discussed in this review was interpreted in the context of ClinPGx clinical annotation levels of evidence, which classify gene–drug associations from Level 1A (highest evidence with guideline or replicated clinical support) to Level 4 (limited or preliminary evidence). 21​

**Table:** **Table 1** Examples of PGx implications in medications commonly used by athletes

Drugs	Primary metabolizing enzymes	Potential clinical implications	PGx guided recommendations
SSRIs			
Fluoxetine 48​ ​49​	CYP2D6	Altered metabolism may affect serum concentrations, tolerability, and risk of adverse effects	N/A. Current evidence is insufficient for formal CPIC or DPWG dosing recommendations.
Paroxetine 48​ ​49​	CYP2D6	Increased serum concentrations and risk of adverse effects in PMs and reduced efficacy in UMs	PM: Consider a 50% reduction in the recommended standard dose, slower titration schedule, and a 50% lower maintenance dose. IM: Consider a lower starting dose and slower titration schedule. UM: Select an alternative drug not predominantly metabolized by CYP2D6
Citalopram 48​ ​49​	CYP2C19	Increased exposure and QT risk in PMs and reduced efficacy in UMs	PM: Consider an alternative drug not predominantly metabolized by CYP2C19. If the drug is clinically appropriate, consider a lower starting dose, a slower titration schedule, and a 50% reduction of the standard maintenance dose. IM: Initiate therapy with the recommended starting dose. Consider a slower titration schedule and lower maintenance. UM: Consider another antidepressant not predominantly metabolized by CYP2C19. If the drug is clinically appropriate, consider titrating to a higher maintenance dose if adequate efficacy is not met.
Escitalopram 48​ ​49​	CYP2C19	Increased exposure and QT risk in PMs and reduced efficacy in UMs	PM: Consider an alternative drug not predominantly metabolized by CYP2C19. If the drug is clinically appropriate, consider a lower starting dose, a slower titration schedule, and a 50% reduction of the standard maintenance dose. IM: Initiate therapy with the recommended starting dose. Consider a slower titration schedule and lower maintenance. UM: Consider another antidepressant not predominantly metabolized by CYP2C19. If the drug is clinically appropriate, consider titrating to a higher maintenance dose if adequate efficacy is not met.
Sertraline 48​ ​49​	CYP2C19	Altered exposure may affect tolerability and efficacy	PM: Consider a lower starting dose, a slower titration schedule, and a 50% reduction in standard maintenance dose, or select a clinically appropriate alternative antidepressant not predominantly metabolized by CYP2C19. DPWG recommends not to give doses exceeding 75 mg/d IM: Initiate therapy with the recommended starting dose. Consider a slower titration schedule and a lower maintenance dose. UM: Initiate therapy with the recommended dose.
SNRIs			
Venlafaxine 49​ ​53​	CYP2D6	PMs may experience higher venlafaxine concentrations and an increased risk of adverse effects due to the reduced conversion to the active metabolite O-desmethylvenlafaxine.	PM: Risk for high drug concentrations and side effects. Consider an alternative antidepressant not predominantly metabolized by CYP2D6. UM: No recommendation.
Opioids			
Codeine 15​ ​39​	CYP2D6	PMs may experience reduced analgesia; UMs may have increased morphine toxicity risk	PM: Significantly less morphine (active metabolite) formation than expected, leading to diminished effects. Avoid codeine or use a non-tramadol opioid. IM: Less morphine (active metabolite) formation than expected. Switch to a non-tramadol opioid if an inadequate response is observed. UM: Increased formation of morphine (active metabolites) leading to a higher risk of toxicity. Avoid codeine, but if an opioid is necessary, use a non-tramadol opioid.
Tramadol 15​ ​39​	CYP2D6	Altered active metabolite formation affects analgesia and toxicity risk	PM: Significantly less O-desmethyltramadol (active metabolite) formation than expected, leading to diminished effects. Avoid tramadol and use an alternative analgesic not affected by the CYP2D6 metabolism IM: Less O-desmethyltramadol (active metabolite) formation than expected. Switch to a non-codeine opioid if an inadequate response is observed. UM: Increased formation of O-desmethyltramadol (active metabolite) leading to a higher risk of toxicity. Avoid tramadol, but if an opioid is necessary, use a non-codeine opioid.
Hydrocodone 15​	CYP2D6	Reduced hydromorphone formation may reduce efficacy	PM: Decreased formation of hydrocodone to hydromorphone (active metabolite), but there is insufficient evidence to determine if these effects interfere with drug efficacy. If no response and opioid use is warranted, consider an alternative opioid.
Oxycodone 15​ ​39​	CYP2D6	Reduced conversion of oxycodone to oxymorphone (active metabolite) in PMs may alter analgesic response	PM: Insufficient evidence to determine if these effects interfere with drug efficacy.
NSAIDs			
Ibuprofen 30​	CYP2C9	Reduced metabolism may increase toxicity risk and drug exposure	PM: Higher concentrations of ibuprofen expected. Start with 25–50% of the lowest recommended dose. Use the lowest effective dose for the shortest duration needed.
Meloxicam 30​	CYP2C9	Increased exposure and prolonged half-life in PMs	PM: Higher concentrations of meloxicam expected. Choose an alternative drug not metabolized by CYP2C9 or an NSAID with a shorter half-life (e.g., ibuprofen)
Celecoxib 30​	CYP2C9	Increased serum concentrations may increase adverse event risk	PM: Higher concentrations of celecoxib expected. Start with 25-50% of the maximum recommended dose. Use the lowest effective dose for the shortest duration needed.

Abbreviations: CPIC, Clinical Pharmacogenetics Implementation Consortium; DPWG, Dutch Pharmacogenetics Working Group; IM, intermediate metabolizer; NM, normal metabolizer; NSAIDs, nonsteroidal anti-inflammatory drugs; PGx, pharmacogenomics; PM, poor metabolizer; SNRIs, serotonin–norepinephrine reuptake inhibitors; SSRIs, selective serotonin reuptake inhibitors; UM, Ultrarapid metabolizer

Note: Examples of PGx implications and genotype-guided prescribing recommendations for medications commonly used by athletes. Recommendations are adapted from CPIC and DPWG guidance where available and are intended to illustrate clinically relevant PGx considerations rather than provide comprehensive prescribing guidance.

## Pharmacogenomics of commonly used medications

Although athletes are generally perceived as healthy individuals, they experience many of the same acute and chronic medical conditions as the general population and often require pharmacological therapy. Physicians caring for athletes should understand the medications they use, including how these substances may interact with performance, exercise, environment, and other drugs. PGx can help stratify the risk of certain medications and personalize treatment. Drug classes commonly used by athletes that have potential PGx implications include SSRIs, SNRIs, opioids, NSAIDs, and even widely consumed substances such as caffeine. 7​ 22​​ ​23​ Each of these drug classes is subject to influence by genetic variations that affect metabolism, efficacy, therapeutic response, and side-effect profiles ( Table 1 ).

### Pain medications

Athletes routinely face musculoskeletal sprains and strains, acute injuries, and chronic overuse conditions due to the physical demands of training and competition. 18​ Whenever possible, non-pharmacological pain management strategies are preferred to minimize medication-related risks. However, when used appropriately, pharmacological options can be safe and effective while improving an athlete’s comfort, function, and quality of life. 19​

#### Nonsteroidal anti-inflammatory drugs

NSAIDs are commonly used across all levels of sports to manage pain and inflammation. These medications are among the most frequently prescribed drug classes for pain management, with millions of prescriptions written annually and billions spent on prescription and over-the-counter formulations. 24​ Data collected from doping control forms indicated that between 16.7 and 54.2% of professional football players use NSAIDs. 22​ According to the International Olympic Committee (IOC) consensus statement on pain management in elite athletes, oral NSAIDs are considered among the preferred pharmacological options for managing mild to moderate acute pain in athletic populations. 19​ Their widespread availability and effectiveness make NSAIDs a staple in athletic care; however, concerns are growing regarding adverse effects and variability in individual responses. 25​ 26​ ​27​

NSAIDs are metabolized in the liver primarily via the CYP2C9 enzyme, with some CYP2C8 involvement. 27​ 28​ ​29​ The function of CYP2C9 declines with the CYP2C9*2,*3, and *13 alleles in combination with any of the three allele types. 30​ CYP2C9*2 exhibits decreased functions while alleles *3 and *13 exhibit no functions. 30​ CYP2C9 PMs and IMs are at increased risk of elevated systemic drug concentrations due to reduced enzymatic clearance. This accumulation may result in ADRs or toxicity. Common NSAID-related side effects include stomach pain and gastrointestinal discomfort such as dyspepsia, nausea, and heartburn. More serious complications may involve gastric ulcers or upper gastrointestinal bleeding.

Athletes using celecoxib and having certain CYP2C9 variants (e.g.,*2,*3) have a higher risk of toxicity via gastrointestinal bleeding than those with CYP2C9*1/*1. 31​ ​32​ A similar association is observed with meloxicam, which is also metabolized primarily by CYP2C9. A decreased function of these variants in metabolizing enzymes requires careful consideration of initial dosing strategies in athletes, including starting athletes on a lower dose or selecting an alternative medication or medication class for pain management. Ibuprofen is predominantly metabolized by CYP2C9 and CYP2C8, and a higher risk of drug accumulation exists in patients with decreased functions of both enzymes. 30​ Ibuprofen toxicity includes seizures, apnea, and hypertension, as well as renal and hepatic functional decline. 33​ PMs (*2/*3 and*3/*3) require 25 to 50% of the lowest recommended starting dose when initiating therapy and IMs (*1/*2,*2/*2,*1/*3) require the lowest recommended starting dose when initiating therapy (Level 1A). Due to the differences in metabolism influenced by the alleles expressed, the ibuprofen dosage should be adjusted accordingly. 30​ ​34​

Unlike most NSAIDs, where decreased CYP2C9 activity is commonly associated with increased toxicity risks, evidence on ketoprofen suggests a different PGx profile. Patients carrying the CYP2C9*3 allele have demonstrated enhanced analgesic effects from ketoprofen, particularly when paired with a normal function allele (Level 3). 26​ This atypical association suggests that reduced metabolic clearance in some individuals may improve drug efficacy rather than heighten ADRs. However, this response is probably multifactorial, influenced by additional genetic polymorphisms and patient-specific clinical factors.

Beyond CYP2C9, additional genetic factors may contribute to variability in NSAID response. Variants involving CYP2C8 and prostaglandin–endoperoxide synthase genes ( *PTGS1* and *PTGS2* ), which encode cyclooxygenase enzymes, have also been investigated for their potential influence on NSAID metabolism, efficacy, and toxicity. 30​ There is currently insufficient evidence to support routine clinical implementation or genotype-guided dosing recommendations for these variants. For athletes who require rapid return to training, the reduced CYP2C9 activity may increase the risk of dose-related adverse effects, making PGx-guided dosing particularly relevant.

NSAID use during prolonged endurance exercise is a modifiable contributor to renal stress or acute kidney injury (AKI) because cyclooxygenase inhibition may impair prostaglandin-mediated preservation of renal perfusion. In athletes undertaking prolonged endurance exercise, NSAID use may compound the renal hypoperfusion due to exercise factors such as exertional heat stress, dehydration, and sympathetic activation, thereby increasing susceptibility to AKI. A 2024 scoping review found that the endurance-sport NSAID literature remains limited and largely observational, but identified recurring concerns about renal injury and other adverse outcomes in marathon and ultra-endurance settings. 35​ Because CYP2C9 decreased-function phenotypes increase exposure to several NSAIDs and have actionable CPIC dosing recommendations, pre-emptive PGx testing may help inform agent selection and dosing in athletes with recurrent NSAID requirements or elevated renal-risk profiles. 30​

#### Opioids

Athletes face elevated risks of acute and chronic musculoskeletal injuries, often managed with an array of analgesics. The IOC consensus statement on pain management in elite athletes notes that opioids are the most effective pharmacological agents for severe acute pain when used in a dose-dependent manner, although their use must be balanced against the potential risks of adverse effects and misuse. 19​ The prevailing culture of playing through pain, at all levels, contributes to opioid misuse vulnerability. Research shows that 4.4 to 4.7% of professional athletes report current use, while 52% of retired NFL players used opioids during their careers, with a striking 71% misusing them. 36​ Opioids work through central and peripheral mechanisms, offering potent analgesia but also a high risk of ADRs. These range from mild symptoms such as constipation, drowsiness, nausea, and dizziness to more serious complications such as respiratory depression, dependence, addiction, and overdose. PGx offers a precision-based strategy to improve opioid prescribing and reduce these risks. 37​ ​38​ The most clinically actionable gene in opioid PGx is CYP2D6, which metabolizes many commonly prescribed opioids, including codeine, tramadol, oxycodone, and hydrocodone. 37​ Notably, codeine and tramadol are prodrugs requiring bioactivation by CYP2D6 to exert analgesic effects. Individuals classified as PM phenotypes exhibit reduced enzymatic activity, leading to minimal or no conversion to the active metabolite and thus inadequate pain control. Conversely, UMs with gene duplication of functional alleles such as*1/*1xN or*2/*2xN convert these prodrugs into their active metabolites at an accelerated rate. This can result in supratherapeutic concentrations and increase the risk of opioid toxicity, including the life-threatening ADR of respiratory depression. Consequently, CPIC and DPWG guidelines recommend avoiding codeine and tramadol for pain management in PMs and UMs due to concerns over therapeutic inefficacy or heightened opioid toxicity, respectively (Level 1A). 15​ ​39​ Actionable PGx labeling recommendations for codeine and CYP2D6 are currently recognized by multiple international regulatory and health agencies, including the United States Food and Drug Administration, Health Canada/Santé Canada, Pharmaceuticals and Medical Devices Agency of Japan, and Swiss Agency for Therapeutic Products (Swissmedic).

Additionally, phenoconversion, where a patient’s actual drug metabolism differs from their genetic prediction, can further complicate genotype-based prescribing. For example, a patient with a genetically normal CYP2D6 phenotype taking a strong CYP2D6 inhibitor, such as fluoxetine, may functionally resemble a PM. This is relevant in athletic populations, where SSRIs such as fluoxetine are commonly prescribed for performance-related anxiety or depression. In this example, CYP2D6 inhibition may reduce opioid efficacy or alter opioid responses. 38​ Incorporating PGx-informed prescribing could enhance opioid selection, mitigate ADRs (e.g., respiratory depression in UMs), improve pain control, and minimize the quantity of unused medication, reducing the risk of diversions or misuse. 37​ ​40​

Additional non-CYP PGx pathways have also been investigated in opioid therapy, including OPRM1, which encodes the μ-opioid receptor, and catechol-O-methyltransferase (COMT), which influences the catecholamine metabolism and pain perception. 15​ Several studies have investigated the OPRM1 A118G polymorphism in relation to opioid response, with evidence suggesting that G-allele carriers may require higher opioid doses to achieve adequate analgesia. 41​ Although these variants have demonstrated potential associations with opioid response and dose requirements, current evidence remains insufficient to support routine genotype-guided opioid prescribing based on these markers. 15​ Clinical testing is available for OPRM1 and COMT, but there are no therapeutic recommendations based on results.

### Mental health

Athletes represent a distinct population with an increased vulnerability to mental health challenges stemming from the intense demands and pressures of competitive sport. 42​ The constant pursuit of peak performance, social media and public scrutiny, and pressure to maintain a competitive status can contribute to a range of mental health concerns such as anxiety, depression, and burnout. Furthermore, the peak competitive years for elite athletes tend to overlap with the peak age for the risk of onset of mental disorders. 43​

#### Selective serotonin reuptake inhibitors

As mental health awareness expands and the social stigma around it decreases within the athletic community, SSRIs are increasingly prevalent in managing performance-related mental health conditions, particularly anxiety and depression. 20​ SSRIs are a fundamental pharmacological treatment for numerous psychiatric disorders but are primarily used to manage depression and anxiety. 44​ The IOC on mental health in elite athletes notes that SSRIs are commonly prescribed to manage depression in athletes, with fluoxetine specifically emerging as a preferred option due to the lack of demonstrated negative effects on athletic performance. 20​ In the context of sports and PGx insights into SSRI metabolism, leveraging established PGx profiles of commonly used SSRIs enables clinicians to individualize an athlete’s therapy. Integrating PGx testing into mental healthcare for athletes holds promise for improving psychological well-being and competitive longevity.

Fluoxetine is primarily metabolized by the CYP2D6 enzyme, which converts the drug to its active metabolite of ( *S* )-norfluoxetine. 45​ The *CYP2D* 6 gene is highly polymorphic, and numerous allele variants significantly influence fluoxetine PK. Individuals classified as PMs or IMs exhibit the reduced activity of the CYP2D6 enzyme. 46​ The allele variants CYP2D6*3, CYP2D6*4, CYP2D6*5, and CYP2D6*6 are responsible for the reduced gene activity. Individuals possessing these variants exhibit higher fluoxetine/(S)-norfluoxetine plasma ratios, suggesting impaired conversion to the active metabolite and potentially diminished therapeutic efficacy (Level 3).

Conversely, RMs and UMs possess one or more fully functional alleles, leading to normal or increased enzymatic activity. For UMs, higher enzymatic activity results in increased conversion to ( *S* )-norfluoxetine, which may lead to enhanced therapeutic efficacy or an elevated risk of dose-dependent ADRs, such as agitation, insomnia, and gastrointestinal disturbances (Level 3). The clinical impact of the increased enzymatic activity depends on the number of functioning alleles present. Understanding these PGx distinctions is essential to guide SSRI dosing strategies and mitigate side-effect burdens. However, current evidence from CPIC and DPWG does not support clinically actionable PGx dosing recommendations for fluoxetine. 47​ ​48​

Citalopram and escitalopram, the therapeutically active *S* -enantiomer of citalopram, are metabolized by CYP2C19. Variations in CYP2C19 can influence their PK and thus effectiveness and tolerability. PMs, characterized by diplotypes CYP2C19*2/*2,*2/*3, and*3/*3, exhibit reduced enzymatic activity, leading to elevated plasma concentrations and heightened the risk of ADRs. 49​ The increased exposure could lead to ADRs such as QT interval prolongation, sedation, and gastrointestinal symptoms (Level 1A). In these patients, a dose reduction or an alternative SSRI not primarily metabolized by CYP2C19 could be considered. IMs also demonstrate the reduced enzymatic function but do not have a markedly higher risk of experiencing adverse side effects. NMs represent the reference group, often tolerating standard dosing regimens. Conversely, UMs possess increased CYP2C19 enzymatic function, resulting in accelerated clearance and subtherapeutic plasma concentrations, increasing the probability of treatment nonresponse. In these cases, clinicians may consider an alternative medication not metabolized by CYP2C19 or adjustment to a higher maintenance dose, while closely monitoring clinical responses. Because untreated anxiety and depression can impair both performance and recovery, optimizing antidepressant therapy while minimizing adverse effects is particularly relevant in athletes.

Beyond CYP450 metabolism, PD variants involving the serotonin transporter gene *SLC6A* 4 and the serotonin receptor gene *HTR2A* have also been investigated for their potential influence on SSRI efficacy, remission, and adverse effect profiles. 49​ However, current evidence remains inconsistent and insufficient to support routine genotype-guided SSRI prescribing based on these variants. In addition to traditional PK gene–drug interactions, inherited arrhythmia susceptibility variants also represent an important consideration in athlete medication management. Certain SSRIs, particularly citalopram and fluvoxamine, have been associated with QT prolongation risk through effects on cardiac ion channels, including hERG/KCNH2 pathways. 50​ Athletes with underlying genetic predispositions affecting cardiac conduction or QT interval regulation could face an increased risk of adverse cardiac events when prescribed medications, such as SSRIs, that can increase the QT prolongation risk. 50​ ​51​

#### Serotonin–norepinephrine reuptake inhibitors

SNRIs may be used for mood stabilization and the management of musculoskeletal or neuropathic pain. However, SNRIs are associated with a greater risk of elevated blood pressure compared to SSRIs. 52​ Venlafaxine primarily undergoes metabolism by CYP2D6 to form the active metabolite O-desmethylvenlafaxine (desvenlafaxine). Individuals with CYP2D6 PM status exhibit reduced conversion to the active metabolite and thus may experience decreased therapeutic efficacy (Level 1A). 53​ In these cases, clinicians should consider a more clinically appropriate medication not primarily metabolized by CYP2D6. The genotype may also influence the cardiotoxicity risk of venlafaxine. A case study highlighted a potential correlation between cardiotoxicity and drug metabolism in reversible cardiac dysfunction observed after saturation of O-desmethylvenlafaxine after venlafaxine poisoning in a patient who was a CYP2D6 RM and a CYP2C19 UM. 54​

#### Caffeine

Caffeine is among the most widely used ergogenic aids in sports, with robust evidence supporting performance-enhancing effects on physical and cognitive domains. Research has consistently demonstrated improvement in muscular endurance, strength, sprinting capacity, and aerobic performance across a broad range of athletic disciplines. 55​ These benefits are typically observed with doses between 3 and 6 mg/kg body weight, although individual variability in response is substantial. This wide variability in response is influenced by genetic factors, habitual consumption, and caffeine sources. All these factors affect dose consideration when tailoring caffeine use in athletes. Caffeine exerts its stimulant effects through adenosine receptor antagonism in the brain, resulting in positive inotropic and chronotropic effects, vasodilation, and cardiac contractility. Caffeine is a CYP1A2 substrate, and CYP1A2 variations can influence an individual’s sensitivity and ADR risks from caffeine (Level 3). 17​ For example, CYP1A2*1F carriers exhibit increased enzyme activity, often requiring higher doses to achieve similar ergogenic effects to an NM. Individuals with variations in their A1 and A2a adenosine receptor genes may experience enhanced caffeine sensitivity, increasing their susceptibility to potential ADRs such as hypertension, tachycardia, insomnia, and anxiety. 56​ For female athletes, the use of oral contraceptives and hormone replacement therapy must be considered when evaluating CYP1A2 status, as these act as CYP1A2 inhibitors, potentially leading to slower caffeine clearance and enhanced side-effect profiles. 17​

## Discussion

In optimizing athlete health, safety, and performance, PGx has the potential to become an important component of athlete healthcare. The wide range of PGx implications and gene–drug interactions associated with commonly prescribed medications in athletes underscores the need to shift from reactive prescribing to a proactive, genetically individualized approach. Broad-panel PGx testing, as opposed to single-gene or reactive testing, offers a comprehensive and proactive strategy for athlete healthcare. Broad-panel testing screens for multiple clinically actionable gene variants simultaneously, equipping clinicians with insights to tailor therapy for not only current medications but also future prescribing decisions ( Fig. 3 ). Compared to reactive testing, proactive broad-panel PGx testing may reduce the need for redundant testing, decrease trial-and-error prescribing, and minimize treatment delays. For athletic populations, where the smallest gains matter, ADRs can derail careers, and the therapeutic window is narrow, PGx testing is an important tool for clinicians to utilize.

**Fig. 3:**
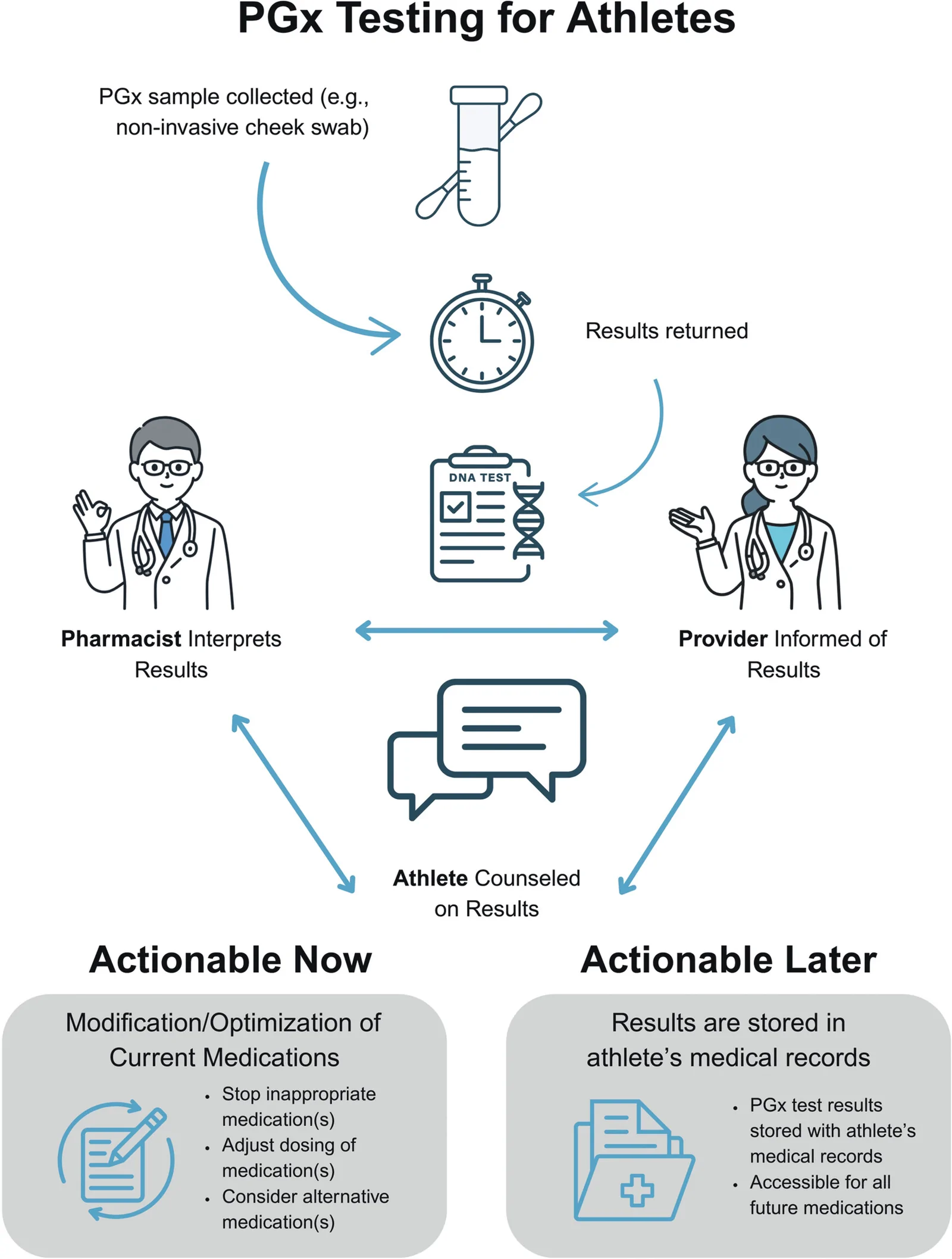
Pharmacogenomic testing for athletes: PGx testing for athletes begins with a non-invasive cheek swab, with results available in approximately 2 weeks. A pharmacist interprets the results, which are shared with the provider and the athlete. This enables immediate medication optimization, such as discontinuing, adjusting, or selecting alternative therapies. It also enables results to be stored in the athlete’s medical record for future prescribing decisions.

Proactive PGx testing enables providers to integrate genetic insights into decision-making before adverse events or therapeutic failures occur, enhancing performance outcomes and athlete safety. As the medication use in sports continues to grow, integrating genetic insights into prescribing may advance precision athlete care. However, successful implementation hinges on having not only the testing results but also the appropriate expertise. A significant barrier healthcare providers often cite is insufficient PGx training, which leads to underutilization even when testing is available. 57​ Bridging this gap requires education, interdisciplinary collaboration, and the inclusion of PGx-trained clinicians in athlete healthcare teams. Pharmacists are recognized PGx experts and are uniquely positioned to lead the interpretation and application of PGx testing in clinical settings. 58​ Due to their focused training in drug–gene interactions, PK, and therapeutic optimization, pharmacists are well equipped to translate genetic test results into actionable patient-specific recommendations. These may include adjusting medication selection, dosing, or monitoring parameters. CPIC specifically recommends that PGx testing be guided and interpreted by healthcare professionals with PGx expertise, and pharmacists are often identified as the most qualified professionals for this role. 3​ In athletic settings, pharmacists can tailor individualized therapy recommendations, considering genetic variability and sport-specific demands, such as training recovery and anti-doping compliance. PGx-trained pharmacists may play an important role on athlete healthcare teams. These updated athlete care models provide a proactive, evidence-based approach to medication management that supports athlete health, safety, and long-term performance.

One of the most significant limitations in the current literature and this review is the lack of studies investigating the impact of PGx interventions, specifically on athletic performance or recovery outcomes. While PGx data offer insight into safer and more effective medication use, no clinical trials have yet evaluated whether these interventions directly translate to improved athletic performance or recovery metrics, injury recovery times, or training adaptations. Thus, our recommendations are extrapolated from general clinical PGx evidence applied to the unique context of athletes. Additionally, much of the currently available PGx evidence has been generated from general clinical populations rather than athlete-specific cohorts, which may limit direct extrapolation to athletes due to differences in physiology, training demands, recovery patterns, and medication utilization.

Another limitation is that this review relied heavily on CPIC guidelines and ClinPGx data, which may not capture all the emerging gene–drug interactions or nuances in athletic populations. Finally, while our selection of medications was grounded in common use among athletes, it was not exhaustive and may have excluded niche therapies relevant in specific sports or regions that could benefit from a PGx-guided approach.

Widespread PGx implementation faces barriers such as limited clinician education, the need for robust clinical and cost-effectiveness data, and ethical concerns regarding privacy and access. 38​ Several studies have demonstrated that many healthcare providers report low confidence in ordering, interpreting, and applying PGx results in clinical practice. 59​ ​60​ Many healthcare institutions also lack the infrastructure to support integration, such as electronic health records equipped with clinical decision support tools and standardized billing processes for genetic services. 57​ Moreover, institutional uncertainty about long-term cost-effectiveness continues to slow progress, despite evidence suggesting downstream savings from optimized prescribing and reduced ADRs. 57​ ​61​

Ethical considerations around PGx testing in athletes extend beyond standard clinical consent because genetic information may have implications beyond direct patient care. In sport settings, concerns include privacy, team selection, contract decisions, insurance, and how coaches, medical staff, teams, and sport organizations may access or use genetic information. 62​ ​63​ A medication-safety test can be ethically defensible when it answers a real prescribing question such as reducing exposure to a drug in a patient with a well-established reduced-metabolism phenotype. The Australian Institute of Sport position statement states that the proposed use of PGx is therapeutic and medication-safety oriented, not predictive performance genomics. 62​

Results should not be extended beyond their intended clinical purpose or used for performance prediction, talent identification, injury-risk assessment, or athlete selection. Programs should also provide access to PGx or genetic-counseling expertise when results are complex or sequencing-based. Ethical considerations may also vary by testing platform. Targeted genotyping approaches such as MassARRAY generally test predefined variants, whereas next-generation sequencing can generate broader genomic information and potentially identify findings beyond the original prescribing question. 64​ Broader genomic testing may require additional consideration of consent, privacy, and incidental findings.

Future research should focus on assessing the clinical utility of PGx testing within athletic populations. For example, a prospective study could compare recovery times among athletes prescribed postoperative analgesics, with one group receiving PGx-guided therapy and the other receiving standard care without prior genetic testing. Prospective studies must evaluate whether PGx-guided decisions lead to measurable improvements in outcomes important to athletes. Future research should explore athlete-specific barriers to PGx adoption, including accessibility and concerns about genetic privacy.

## Conclusion

PGx, the study of how genetic variations influence drug responses, has transformed the landscape of individualized medicine by enabling clinicians to match therapies to individual genetic profiles. Its clinical utility is well-established across multiple specialties, guiding safer and more effective prescribing practices. Its application remains largely untapped in sports medicine practices. Athletes frequently use medications, such as NSAIDs, opioids, SSRIs, and caffeine, carrying well-documented gene–drug interactions. Genetic differences in major drug-metabolizing enzymes such as CYP2C9, CYP2D6, and CYP2C19 can influence how these medications are processed, impacting therapeutic efficacy, side-effect risk, performance, and recovery outcomes. Integrating PGx testing into athlete care can provide critical insights for enabling more informed prescribing and optimized medication selection and dosing, while considering the unique demands of the athlete and their performance.

## Disclaimer

The views expressed in this material are those of the authors and do not reflect the official policy or position of the Larkin University or the Sports Pharmacy Network.

## Declaration of GenAI Use

The authors confirm that no generative artificial intelligence was used in the preparation of this manuscript.
